# Gender and education differences in sedentary behaviour in Canada: an analysis of national cross-sectional surveys

**DOI:** 10.1186/s12889-020-09234-y

**Published:** 2020-07-27

**Authors:** Stephanie A. Prince, Karen C. Roberts, Alexandria Melvin, Gregory P. Butler, Wendy Thompson

**Affiliations:** 1 Centre for Surveillance and Applied Research, Public Health Agency of Canada, 785 Carling Avenue, Ottawa, Ontario K1A 0K9 Canada; 2 Division of Cardiac Prevention and Rehabilitation, University of Ottawa Heart Institute, Ottawa, Canada

**Keywords:** Sedentary behaviour, Surveillance, Gender, Education

## Abstract

**Background:**

Canadians spend the majority of their days sedentary. Gender and education are important social determinants of health that impact health behaviours. There is evidence that gender and educational differences in sedentary behaviour exist. In Canada, while general trends suggest that leisure sedentary activities have changed; there has been no comprehensive assessment examining whether historical changes in sedentary behaviour differ by gender and education level. Our objective was to examine whether gender and educational differences in accelerometer-measured sedentary time and self-reported sedentary behaviours exist among Canadians and if differences are consistent across age groups, over time and across multiple survey sources.

**Methods:**

We summarize amounts of total accelerometer-measured sedentary time and self-reported sedentary activities (e.g., passive travel, television, computer, video games, screen, reading) by age (i.e. children: 6–11 years, youth: 12–17 years, adults: 18–34 years, 45–49 years, 50–64 years, and older adults: ≥ 65 years), gender (girls/women, boys/men) and household education level (< post-secondary vs. ≥ post-secondary) over time in the Canadian Community Health Survey, Canadian Health Measures Survey, General Social Survey, and the Health Behaviour in School-Aged Children study. Gender and education level differences are examined using independent sample t-tests or chi-square analyses.

**Results:**

While few differences were found for total accelerometer-measured sedentary time, gender and education differences in self-reported, type-specific sedentary behaviour were identified. Among youth, data from all surveys consistently identified that boys engaged in more video/computer game play (e.g., boys: 0.35–2.68 vs. girls: 0.09–2.15 h/day), while girls engaged in more leisure reading (e.g., boys: 0.45–0.65 vs. girls: 0.71–0.99 h/day). Those with a higher education or household education often reported more leisure reading and passive travel. Education level differences in screen time were often age dependent, with leisure computer use greater in higher education groups in adults only and leisure television watching generally higher in lower education groups in children and adults, but not youth.

**Conclusions:**

This information is valuable as it helps to identify segments of the population which may be at greater risk for engaging in higher volumes of sedentary behaviour. In turn, this information can identify target audiences and behaviours for policies and interventions. Future work is needed to further understand factors contributing to these differences (e.g., preferences, occupation, family structure).

## Background

Sedentary behaviour is pervasive in our modern day world [1]. It is not synonymous with inactivity which refers to those who are not physically active enough to gain health benefits (i.e. not meeting physical activity guidelines). Rather sedentary behaviour includes pursuits undertaken at a low energy expenditure (i.e., ≤ 1.5 metabolic equivalents) while in a sitting, lying or reclining position such as watching television, using a computer or motorized commuting [2]. Canadians are largely sedentary and inactive (not meeting physical activity guidelines) [3], placing them at an increased risk for several chronic conditions (e.g., cardiovascular disease, diabetes, obesity, cancer, and depression), lower cognitive development, and mortality [4–12]. Based on this evidence, the Canadian 24-Hour Movement Guidelines for Children and Youth recommend that children and youth limit their leisure screen time to no more than 2 h per day and limit sitting for extended periods [13]. Guidelines for Canadian adults are under development, however, internationally adult guidelines have begun to include recommendations for reducing sedentary behaviour and limiting prolonged periods spent sedentary [14–16].

Understanding the factors that may influence these prevalent behaviours is important for policy and intervention development. Gender and education are important social determinants of health that impact health behaviours [17]. ‘Gender’ and ‘sex’ while often used interchangeably, are separate distinct concepts. An individual’s sex refers to their biological characteristics whereas gender refers to the “socially constructed roles, behaviours, expressions and identities of girls, women, boys, men, and gender diverse people”, it is “usually conceptualized as binary (girl/woman and boy/man), yet there is considerable diversity in how individuals and groups understand, experience, and express it” [18]. Gender may influence sedentary behaviour through socially-constructed norms and roles and can be affected by differential access to resources, opportunities and power [18, 19]. Whereas education (including parental education in the case of children/youth) can greatly influence health behaviours through improved (parental) literacy and knowledge of behaviours and understanding of health messages, access to resources and recreational opportunities, employment opportunities (healthier work conditions, higher compensation and benefits), and exposure to social and psychological factors (i.e., perceived behavioural control, social support, social norms) [17, 20, 21]. Education is often used as a proxy for socio-economic status or social standing. Education and specifically household education, has advantages over other indicators of socio-economic status as it is relatively easy to measure and recall and has higher response rates than income [22]. Consistent evidence has identified that socio-economic status (including education) plays a major role in physical activity levels [23, 24]. While sedentary behaviour research is less established compared to that of physical activity, to date, evidence around correlates of adult sedentary behaviour has found that the association with education depends on the type and domain [25, 26].

Evidence suggests that gender and educational differences in sedentary activities vary across the life course. Among youth, there is evidence that gender and parental education differences in sedentary behaviour exist [27–32]. Boys have been shown to engage in more device-measured total sedentary time, but there is inconsistent evidence on whether gender is associated with screen time [30]. Further, among youth, greater socio-economic status (including higher parental education) is associated with lower leisure screen time and television time [27–29]. Among adults (18–64 years), systematic review evidence suggests that sedentary behaviour and time spent in various sedentary activities differs by gender [26]. Female gender is negatively associated with total sitting, television and screen entertainment and passive travel when compared to male gender [26]. There is no clear association between education and leisure sedentary behaviour, self-reported sitting, or device-measured total sedentary time [25]. Finally, among older adults (≥ 65 years), systematic review evidence has found a lack of consistent association with gender, but greater educational attainment is often negatively associated with time spent sedentary [33].

Internationally, trends in sedentary behaviour have been shown to differ between men and women. In Australia, an analysis of Time Use Surveys between 1997 and 2006 found that total non-occupational sedentary time was found to significantly increase among men, but not women whereas sedentary leisure activities declined in women, but did not change among men [34]. In the United States, data from the 2001 to 2016 National Health and Nutrition Examination Survey found that across all age groups and time, boys reported significantly greater time spent watching television/videos and using a computer outside of school/work [35]. In Canada, while general trends suggest that leisure sedentary activities have changed [36], there has been no comprehensive assessment examining whether historical changes in sedentary behaviour differ by gender and education level. Additionally, while it is clear that different questions and survey sources may yield different population levels of sedentary behaviour [36], it is not clear if gender and education differences in estimates are consistent across these survey sources or if differences have remained over time. Our objective was to examine whether gender and educational differences in accelerometer-measured sedentary time and self-reported sedentary behaviours exist among Canadians and if differences are consistent across age groups, over time and across multiple survey sources.

## Methods

### Study population

The prevalence of sedentary activities by gender and education was examined within four repeated national cross-sectional surveys including the: Canadian Community Health Survey (CCHS); Canadian Health Measures Survey (CHMS); General Social Survey (GSS); and, the Health Behaviour in School Aged Children Survey (HBSC). Only survey years where content is representative at the national level (i.e. core content) are presented.

We examined the sedentary behaviour of children (6–11 years), youth (12–17 years), adults (18–34 years, 45–49 years, 50–64 years), and older adults (≥ 65 years). Within the GSS, only youth aged 15 to 17 years are captured. Within the HBSC, youth are defined as school students in grades 6–11.

### Surveys

#### Canadian Community Health Survey (CCHS)

The CCHS is a nationally representative, cross-sectional health survey of the Canadian household-dwelling population aged 12 years and older. The survey’s coverage excludes: “persons living on reserves and Aboriginal settlements in the provinces; full-time members of the Canadian forces; the institutionalized population; children aged 12-17 that are living in foster care; and persons living in the Quebec regions of Région du Nunavik and Région des Terres-Cries-de-la-Baie-James. Altogether, these exclusions represent less than 3% of the Canadian population aged 12 years and over” [37]. Information is collected in person and by telephone using self-reported questionnaires [37]. Data for this analysis comes from annual cycles (2007, 2008, 2011, 2012, 2017, and 2018), and the Nutrition focus surveys (2004 and 2015).

#### Canadian Health Measures Survey (CHMS)

The CHMS collects self-reported and objectively measured health information from a representative sample of the Canadian household-dwelling population aged 3 to 79 years living in the 10 provinces [38, 39]. The survey’s coverage excludes: “persons living in the three territories; persons living on reserves and other Aboriginal settlements in the provinces; full-time members of the Canadian Forces; the institutionalized population and residents of certain remote regions. Altogether these exclusions represent approximately 4% of the target population” [39]. This analysis uses data from the first five cycles of the CHMS: Cycle 1 (2007–2009), Cycle 2 (2009–2011), Cycle 3 (2012–2013), Cycle 4 (2014–2015), and Cycle 5 (2016–2017). The CHMS collects data from an interview-administered questionnaire conducted in the respondent’s home, as well as from a visit to a mobile examination centre where physical measures are taken. For children aged 3 to 11 years, the parents/guardians answer the household questionnaire on their behalf.

#### General Social Survey (GSS) – Time-use surveys

The GSS collects information on living conditions, social life, and the well-being of Canadians aged 15 and over living in private households in the 10 provinces excluding full-time residents of institutions [40]. Time-use surveys are conducted every 5–7 years and employ a retrospective 24-h time diary to collect information on respondents’ participation in a wide variety of day-to-day activities [41]. This analysis includes all cycles of the GSS with a time-use survey: Cycle 2 (1986), Cycle 7 (1992), Cycle 12 (1998), Cycle 19 (2005), Cycle 24 (2010), and Cycle 29 (2015).

#### Health Behaviour in School Aged Children Survey (HBSC)

The HBSC is a nationally representative survey that collects information on the health and well-being, social environments and health behaviours of school students (grades 6–11; aged 11–15 years) [42–44]. The HBSC is a collaborative study with the World Health Organization Regional Office for Europe with 49 participating countries across Europe and North America. This analysis uses Canadian data from the 1990, 1994, 1998, 2006, 2010, and 2014 surveys.

### Measure of gender

Within all surveys, gender was assessed as either ‘male gender’ or ‘female gender’. As part of the interviewer-administered questionnaire in the CCHS, CHMS and GSS, respondent gender, ‘male’ or ‘female’, was selected by the interviewer. When unsure, the interviewer asked respondents to self-identify as either ‘male’ or ‘female’. In the HBSC, the questionnaire asked whether students were ‘male’ or ‘female’. Important to note is that respondents may have reported gender as sex at birth, whereas others may have reported current/lived gender. Beginning in 2021, the CCHS and CHMS will ask for biological sex at birth and gender, and the GSS will only request gender.

### Measures of education

Within the CCHS and CHMS, household education was assessed by asking respondents to indicate the highest level of education acquired by any member of the household and analyzed as a two-level categorical variable: less than post-secondary graduate compared to post-secondary graduate. Henceforth we use lower and higher household education to describe the two groups. Within the GSS, household education is not assessed, therefore, a two-level categorical variable representing the highest level of educational attainment by the respondent (less than post-secondary graduate vs. post-secondary graduate) was used. Within the HBSC, there is no measure of household education.

### Measures of sedentary behaviour

Due to frequent changes in the questions and/or response options provided to assess sedentary activities over time, there is significant discontinuity in trends. Details of the specific questions/responses used over time within each survey have been compared elsewhere [36].

#### Total accelerometer-measured sedentary time

The CHMS was the only survey that measured total sedentary time using accelerometers. Actical accelerometers (Philips Respironics, Oregon, United States) which record time-stamped acceleration in all directions were worn by ambulatory CHMS respondents. To identify sedentary time, an index of movement intensity based on a count-per-minute value of less than 100 cpm was used [45].

#### Leisure television and video time

Self-reported leisure time spent watching television and videos was examined within the CCHS, CHMS and HBSC. In the CHMS, among children, parental reported time spent watching television or videos or playing video games was assessed by a single question. In the GSS all television watching time was captured, however, given that television is largely viewed as a leisure behaviour, we have included it alongside the other surveys.

#### Leisure computer time

Self-reported leisure time spent using a computer was examined within the CCHS, CHMS and HBSC. Over time, survey questions in all surveys have been modified to adapt to the ever-changing ways in which Canadians interface with technology and e-communication by including the use of other electronic devices (e.g., tablets, smartphones) alongside traditional computer use.

#### Leisure video game time

Self-reported total leisure video game time was examined using data from the CCHS, CHMS (except children) and HBSC. Specific questions on passive (i.e. done while sitting) and active (i.e. physical movement required) video games were included in the CCHS (2011, 2012) and CHMS surveys, however here we exclude active video games.

#### Leisure total screen time

Times spent watching television or videos, using a computer, or other electronic devices were summed within the CCHS, CHMS and HBSC to derive total self-reported leisure screen time. For children and youth, the prevalence meeting the screen-time recommendations from the Canadian 24-Hour Movement Guidelines for Children and Youth (≤ 2 h/day of recreational screen time) [46] was also assessed.

#### Reading time

Leisure time spent reading was assessed by self-report within the CCHS and CHMS. Within the GSS all time spent reading (including online content starting in Cycle 24) was examined.

#### Passive travel time

Passive travel was derived in the GSS and includes combined time spent travelling in a car, bus, taxi, boat/ferry, and/or airplane.

### Statistical analyses

All analyses were conducted using SAS Enterprise Guide v.5.1 and v.7.1 (SAS, Inc., Cary, NC). Means or proportions and 95% confidence intervals (CIs) are presented for the amounts of total sedentary time and specific sedentary activities. These descriptive statistics are presented for each survey, for each gender and education group. Graphs displaying results are presented. Data points are connected if/when questions and response options were consistent between data years. Trends in average daily accelerometer-measured sedentary time by gender and household education across cycles were assessed using unadjusted linear regression analyses. To examine differences between cycles, pairwise contrasts with a Bonferroni adjustment were conducted. To generate continuous measures from questions providing only a categorical response option, we used the midpoint of each category (e.g., 0–1 h = 0.5 h), for the top category we used the starting amount (e.g., more than 20 h = 20 h).

Results are presented by age group. Independent sample t-tests for continuous outcomes and chi-square tests for categorical outcomes were used to assess gender and education group differences.

Survey weights generated by Statistics Canada were used in all analyses of CCHS, CHMS and GSS data to account for the complex survey design, non-response bias and to correctly estimate variance. For CHMS analyses, denominator degrees of freedom were set at 11 for cycles 1, 3, 4 and 5, and 13 for Cycle 2. In the CCHS, CHMS and GSS, the bootstrap technique was used in the estimation of 95% CIs to account for survey design effects. In the first five cycles, the HBSC generated a nationally representative sample that did not require weighting. Analysis of data from 2010 onwards incorporates population weights and controls for clustering at the school level.

## Results

### Accelerometer-measured total sedentary time

**Fig. 1:**
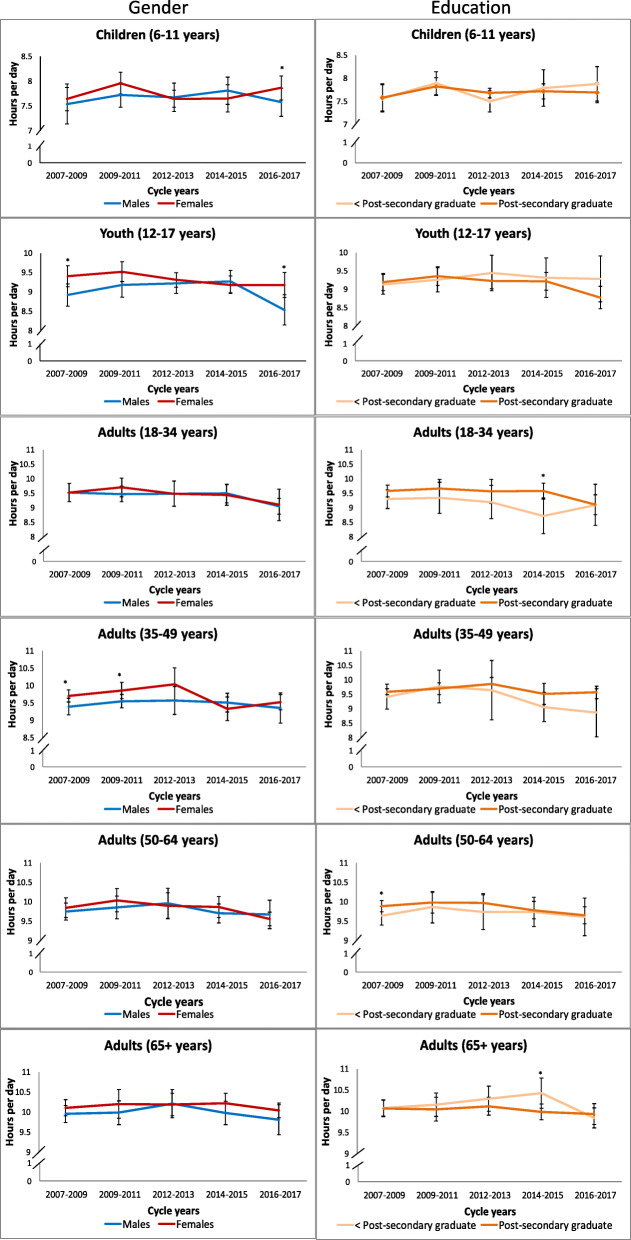
Total accelerometer-measured daily sedentary time by gender and household education level within each age group, 2007–2017. Error bars represent 95% CIs around the mean. Stars (*) indicate differences between education groups at *p* < 0.05. CHMS (2007–2009, 2009–2011, 2012–2013, 2014–2015, 2016–2017)

Figure 1 displays accelerometer-measured total sedentary time within age groups by gender and household education level from the CHMS. Among children, only the most recent cycle (2016–2017) of the CHMS found a significant gender difference, with girls more sedentary than boys (7.9 vs. 7.6 h/day, *p* = 0.045). Similarly, among youth, girls were more sedentary than boys in the earliest (2007–2009; 9.4 vs. 8.9 h/day, *p* = 0.009) and most recent cycles (2016–2017; 9.2 vs. 8.5 h/day, *p* = 0.0035). Among adults, aged 35–49 years, women had significantly higher levels of sedentary time than men in 2007–2009 (9.7 vs. 9.4 h/day, *p* = 0.037) and 2009–2011 (9.9 vs. 9.6 h/day, *p* = 0.03), but not in subsequent cycles. No significant gender differences were found in adults 18–34 years, 50–64 years, or ≥ 65 years. A significant negative linear trend for daily sedentary time in youth boys (β = − 0.08, *p* = 0.047), men aged 18–34 years (β = − 0.09, *p* = 0.04), women aged 35–49 years (β = − 0.09, *p* = 0.007) and women aged 50–64 years (β = − 0.08, *p* = 0.02) was observed with an average decline of ~ 5 min/day per cycle. Pairwise contrasts identified that for male gendered youth, 2012–2013 and 2014–2015 sedentary time was higher than 2016–2017 and among women aged 50–64 years, 2009–2011 sedentary time was higher than 2016–2017.

Very few differences in accelerometer-measured sedentary time by household education were observed among adults, and none were observed among children or youth. In 2014–2015, adults aged 18–34 years from higher education households spent significantly more time sedentary than those from lower education households (9.6 vs. 8.7 h/day, *p* = 0.0098), the opposite was observed in older adults (10.0 vs. 10.4 h/day, *p* = 0.009). In 2007–2009, adults aged 50–64 years living in higher education households, spent significantly more time sedentary than those from lower education households (9.9 vs. 9.7 h/day, *p* = 0.024). Significant negative linear trends were observed for daily sedentary time in higher education households among those aged 12–17 years (β = − 0.11, *p* = 0.006), 18–34 years (β = − 0.10, *p* = 0.02), and 50–64 years (β = − 0.07, *p* = 0.03) with an average decline of ~ 5 min/day per cycle. Pairwise contrasts identified significant between-cycle differences among youth from higher education households (2009–2011 higher than 2016–2017) and adults ≥ 65 years from lower education households (2014–2015 higher than 2016–2017).

### Screen-based sedentary behaviours

#### Watching television or videos

**Fig. 2:**
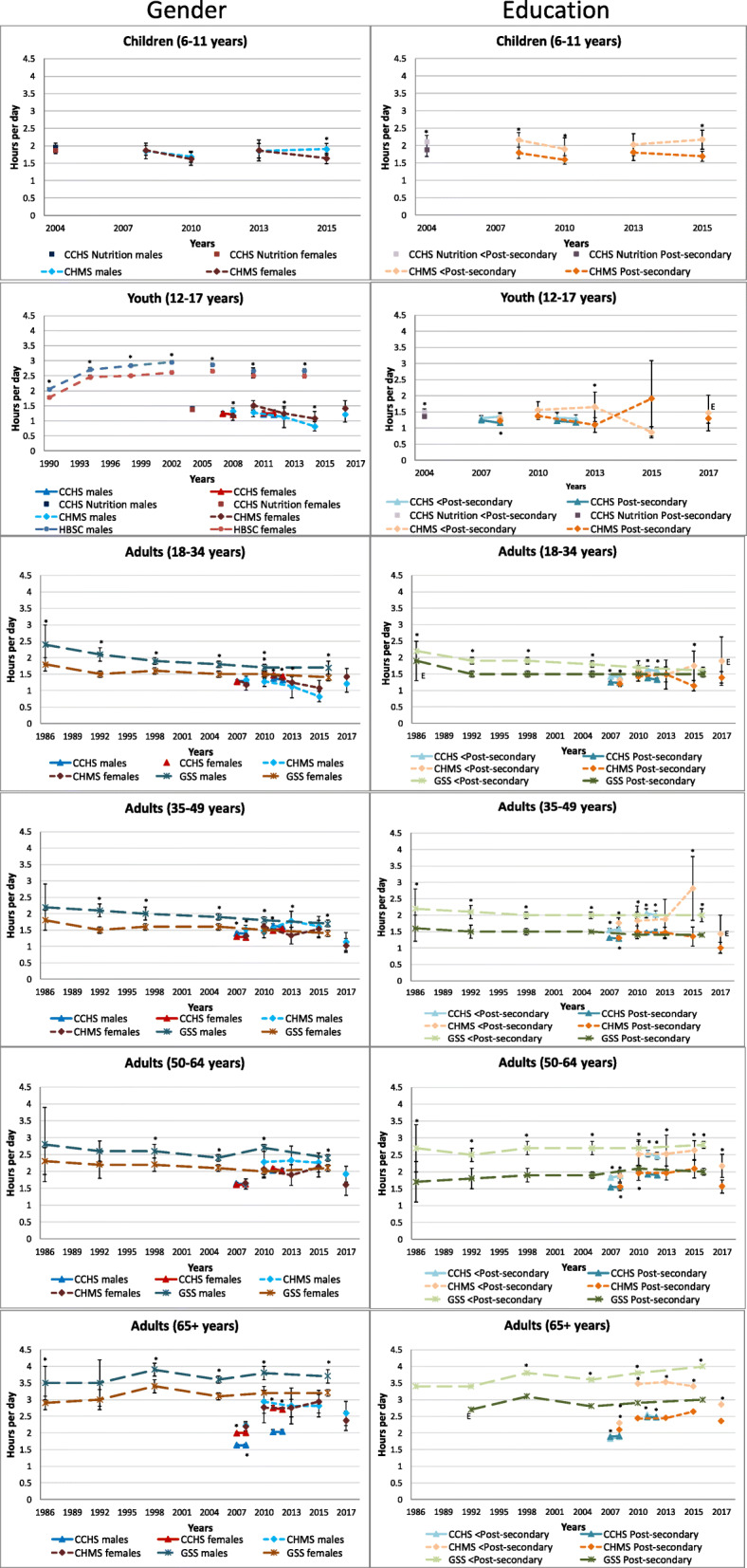
Self-reported daily average leisure television or video watching by gender and household or respondent education within each age group, 1986–2017. Error bars represent 95% CIs around the mean. Stars (*) indicate differences at *p* < 0.05. E – Interpret estimate with caution due to high sampling variability. CCHS Annual – Core content (2007–2008, 2011–2012); CCHS– Nutrition Focus (2004); CHMS (2007–2009, 2009–2011, 2012–2013, 2014–2015, 2016–2017); GSS – Time Use Survey (1986, 1992, 1998, 2005, 2010, 2015); HBSC (1990, 1994, 1998, 2002, 2006, 2010, 2014)

Figure 2 displays self-reported leisure television or video watching time (and video games in children) by gender and education level within age groups. Trends over time in leisure television time appear to be similar between genders. Among children, boys and girls watched similar amounts of television/videos and video games, except in the 2014–2015 CHMS, where boys were found to watch more television than girls (1.9 vs. 1.6 h/day, *p* = 0.002). Among youth, data from the HBSC found that boys reported watching more television than girls at all time points. But data from the CHMS and CCHS either identified no difference, or found that girls reported more television time (depending on survey year). Among adults, regardless of age, the GSS time-use surveys found that men reported higher levels of television viewing than women, while CHMS data rarely detected statistically significant gender differences. Among adults 18–34 years, the 2011 and 2012 CCHS identified women as reporting more television time than men (2011: 1.5 vs. 1.4 h/day; *p* = 0.004, 2012: 1.4 vs. 1.3 h/day; *p* = 0.008). Among adults aged 35–49 years, the 2007, 2008 and 2011 CCHS found that men reported watching significantly more television than women, while this pattern was similar in the 2012 CCHS, it did not reach statistical significance. No differences were found in the CCHS for adults aged 50–64 years. Among older adults, all CCHS data identified that women reported more television viewing than men.

Data from the GSS and CCHS generally identified that trends were similar between education levels, whereas the CHMS found that trends were not always the same in youth and younger adults. Generally, among children, those from lower education households viewed significantly more television than those from higher education households. Among youth, the pattern was less clear and there were few statistically significant differences by education level. Among adults, respondents with lower respondent education level (GSS) or lower household education level (CCHS, CHMS) tended to report more television viewing; the difference was often greater with increasing age.

#### Computer use outside of school or work

**Fig. 3:**
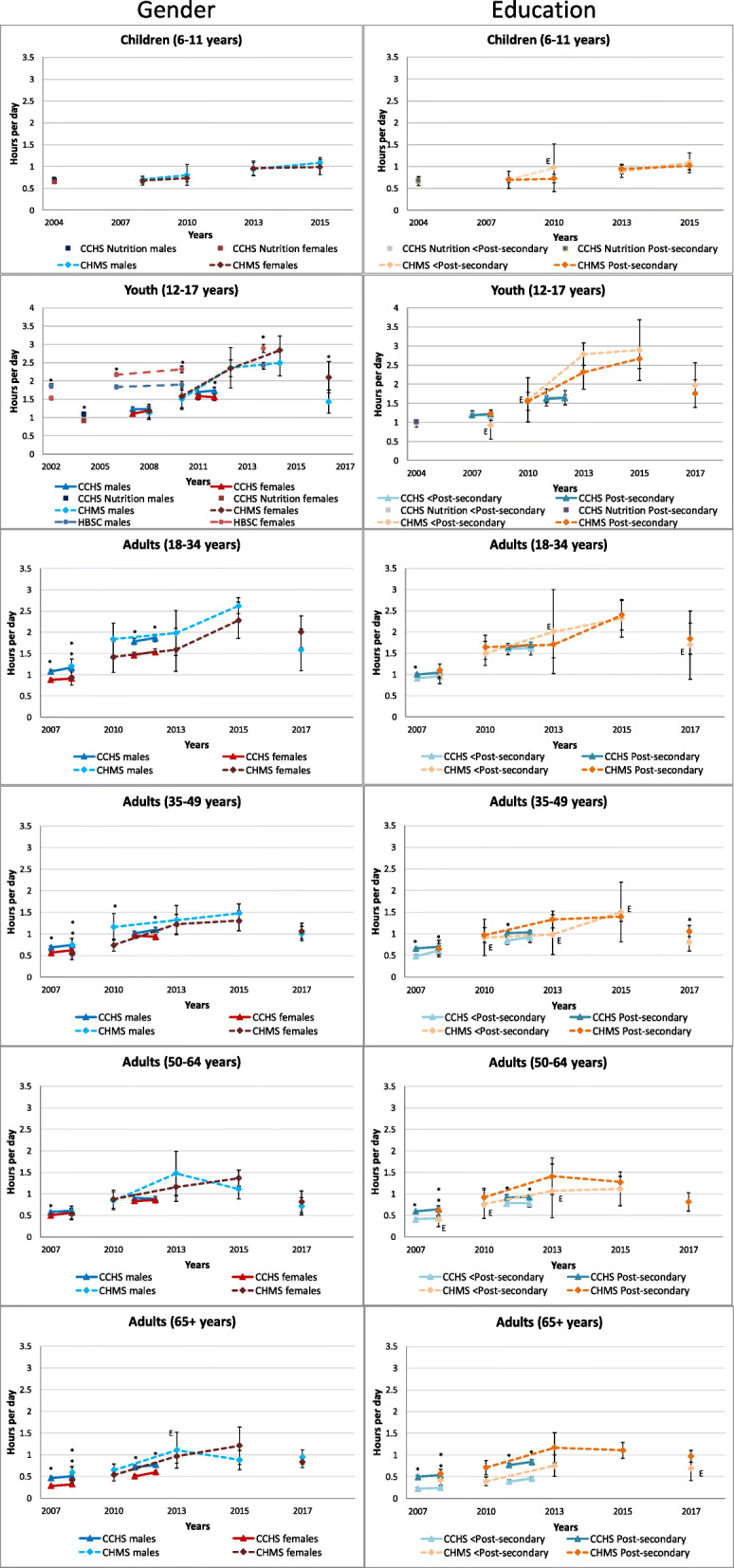
Self-reported daily average leisure computer use by gender and household educational level within each age group, 2002–2017. Error bars represent 95% CIs around the mean. Stars (*) indicate differences at *p* < 0.05. E – Interpret estimate with caution due to high sampling variability. CCHS Annual – Core content (2007–2008, 2011–2012); CCHS – Nutrition Focus Survey (2004); CHMS (2007–2009, 2009–2011, 2012–2013, 2014–2015, 2016–2017); HBSC (1990, 1994, 1998, 2002, 2006, 2010, 2014)

Figure 3 displays parent (children) or self-reported (youth) leisure time spent using a computer by gender and education level within each age group. In general, it appears that trends in leisure computer use time were similar between genders. Among children, no gender differences were observed. Among youth, the HBSC found boys reported more computer use in 2002 (1.9 vs. 1.5 h/day, *p* < .0001), but subsequent years found that girls reported significantly more computer use than boys (e.g., 2014 electronic device time: 2.9 vs. 2.4 h/day, *p* < .0001). Important to note is that more recent HBSC surveys included online chatting time and electronic device time as opposed to ‘computer time’. This is similar to what was observed in the 2016–2017 CHMS which, for the first time, included smart phone use in their leisure computer measure (girls: 2.1 vs. boy: 1.4 h/day, *p* < .0001). Data from the CCHS generally found that youth boys engaged in similar (2007, 2008, and 2011) or significantly higher amounts (2012 and Nutrition 2004) of leisure computer time than youth girls. Among adults aged 18–34 years, 35–49 years (except 2011 CCHS) years and ≥ 65 years, men had significantly higher leisure computer use than women in all years of the CCHS and the earliest cycle of the CHMS. In adults 50–64 years, only the 2007 CCHS found that men self-reported more leisure computer use than women.

Data from the CCHS generally identified that trends in leisure computer use were similar between education levels, whereas they did not always track the same in the CHMS. Among children and youth, no statistically significant differences in leisure computer use were observed by household education levels. Educational differences were apparent with increasing age. The CCHS found that adults aged 35–49, 50–64 and ≥ 65 years, living in higher education households, reported higher levels of leisure computer use than those from lower education households.

#### Video game play

**Fig. 4:**
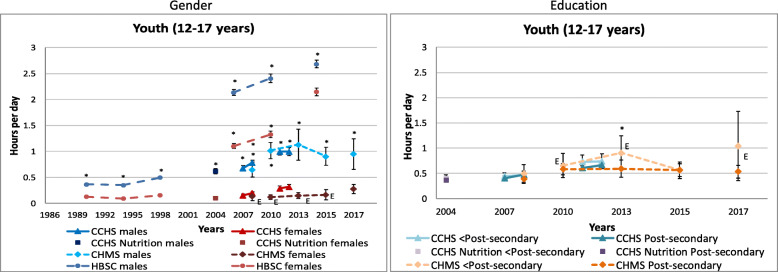
Self-reported daily average video game playing by gender and household education level across survey years in youth**.** Error bars represent 95% CIs around the mean. Stars (*) indicate differences at *p* < 0.05. E – Interpret estimate with caution due to high sampling variability. CCHS Annual – Core content (2007–2008, 2011–2012); CCHS – Nutrition Focus Survey (2004); CHMS (2007–2009, 2009–2011, 2012–2013, 2014–2015, 2016–2017); HBSC (1990, 1994, 1998, 2002, 2006, 2010, 2014)

Figure 4 displays self-reported video game play time in youth by gender and household education level. Boys were consistently found to spend significantly more leisure time playing video games than girls (boys: 0.35–2.68 vs. girls: 0.09–2.15 h/day). Educational differences were only observed in the 2012–2013 CHMS, where those from lower education households reported playing more video games. Among young adults (18–34 years, data not shown), only the 2011 and 2012 CCHS found that those from lower education households reported more time playing video games.

#### Total leisure screen time

**Fig. 5:**
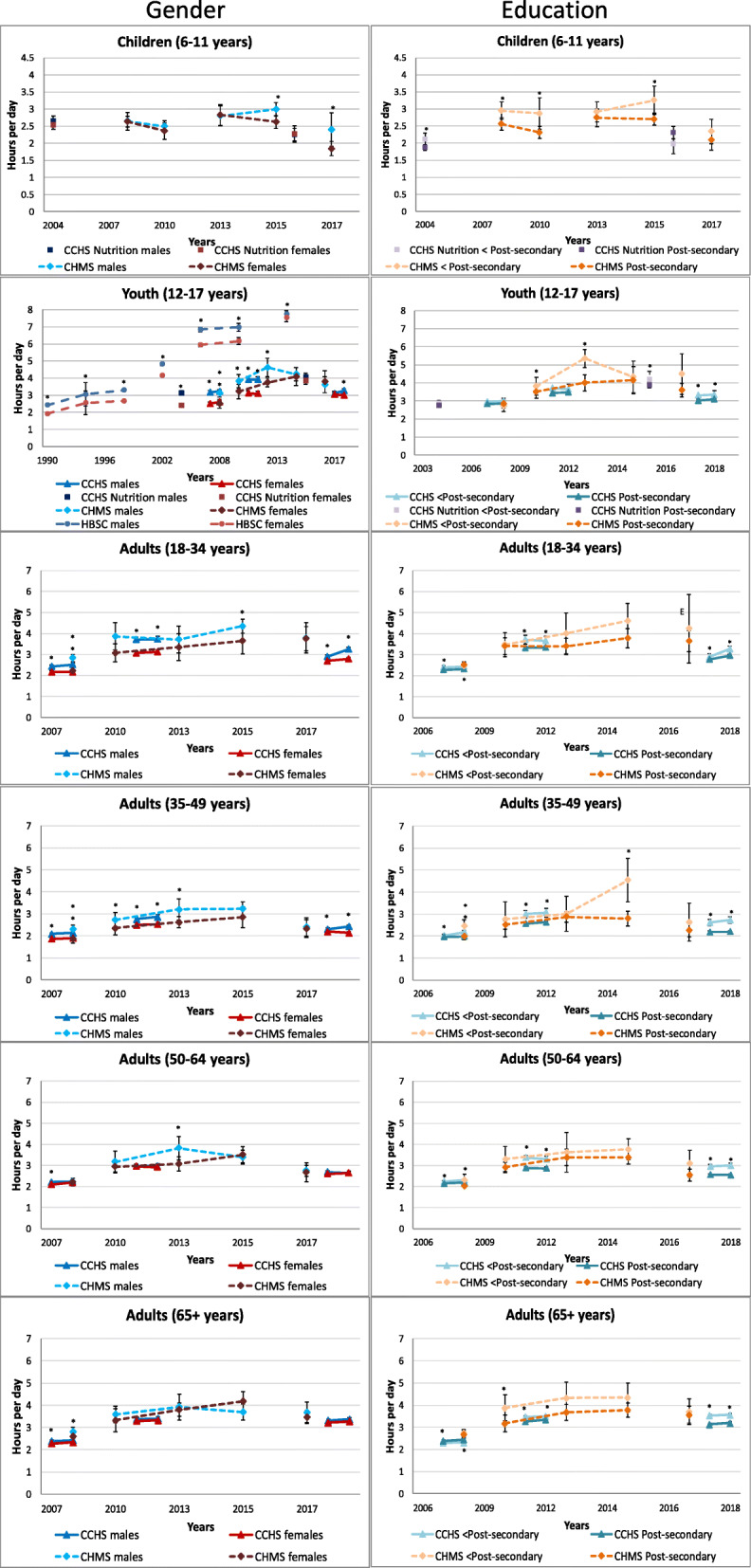
Self-reported daily average leisure screen time by gender and household education level within each age group. Error bars represent 95% confidence intervals around the mean. Stars (*) indicate differences at *p* < 0.05. E – Interpret estimate with caution due to high sampling variability. CCHS Annual – Core content (2007–2008, 2011–2012); CCHS– Nutrition Focus (2004); and CHMS (2007–2009, 2009–2011, 2012–2013, 2014–2015, 2016–2017); HBSC (1990, 1994, 1998, 2002, 2006, 2010, 2014)

Figure 5 displays total parent-reported (children) and self-reported leisure screen time by gender and education level within each age group. Among children, historically boys and girls engaged in similar levels of screen time; however, more recent data from the CHMS shows a gap may be developing with boys spending more leisure time using screens than girls (2016–2017: 2.4 vs. 1.8 h/day, *p* = 0.038). Among youth, across all surveys, boys have generally reported more leisure screen time than girls; however, this gender gap appears to be narrowing with more recent estimates being closer or no longer statistically different (survey dependent). Among adults aged 18–34 and 35–49 years, men historically reported higher levels of leisure screen time than women; however, the gender gap also appears to be narrowing in more recent years. Among adults aged 50–64 years and ≥ 65 years, there have been no consistent differences between men and women.

Among children and youth, those from lower education households generally reported higher levels of leisure screen time, though differences are not always statistically significant. Among adults, a similar pattern is observed, especially in the CCHS, data has consistently demonstrated significant differences in leisure screen time by household education level.

Among children, historically, a similar proportion of boys and girls met the screen time recommendations (≤ 2 h/day) from the Canadian 24-Hour Movement Guidelines [13]. Only in the most recent cycle of the CHMS were girls more likely to meet the recommendation than boys (78.9% vs. 70.0%, *p* = 0.049). Among youth, most data showed that more girls than boys meet the recommendation.

In the 2004 CCHS – Nutrition and the first two cycles of the CHMS, a greater proportion of children from higher education households met the screen time recommendations compared to those from lower education households; this was not seen in more recent years. Among youth, no consistent differences were observed for meeting the screen recommendations by household education level.

### Reading time

**Fig. 6:**
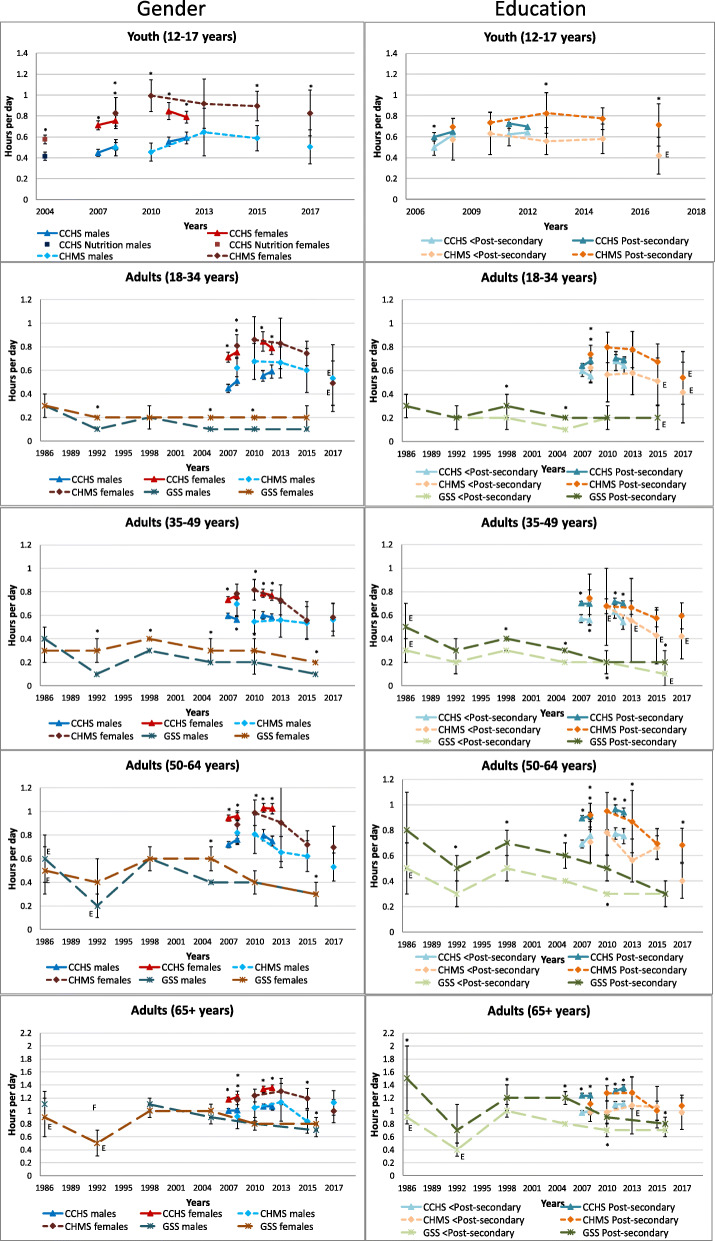
Self-reported daily average leisure reading by gender and household or respondent education level within each age group. Error bars represent 95% confidence intervals around the mean. Stars (*) indicate differences at *p* < 0.05. E – Interpret estimate with caution due to high sampling variability. CCHS Annual – Core content (2007–2008, 2011–2012); CCHS – Nutrition Focus (2004, 2015); CHMS (2007–2009, 2009–2011, 2012–2013, 2014–2015, 2016–2017); GSS – Time Use Survey (1986, 1992, 1998, 2005, 2010, 2015)

Figure 6 displays self-reported reading time by gender and household education level within each age group. Both genders appear to have experienced a decline in leisure reading. Among youth, data from all surveys found that girls engaged in more reading than boys (girls: 0.71–0.99 h/day vs. boys: 0.45–0.65 h/day). The CCHS consistently found that girls/women reported significantly more leisure time spent reading than boys/men regardless of age group; the CHMS found similar differences, though they were not always statistically significant. In the GSS, gender differences in reading time (girls/women reported more time) were observed more consistently amongst younger age groups.

Significant differences between household education levels were less consistently observed for leisure reading time amongst youth and younger adults (18–34 years). Older age groups had more consistent statistically significant differences by education level; those with higher household (CCHS) or respondent education (GSS) tended to engage in more leisure time reading.

### Passive travel

**Fig. 7:**
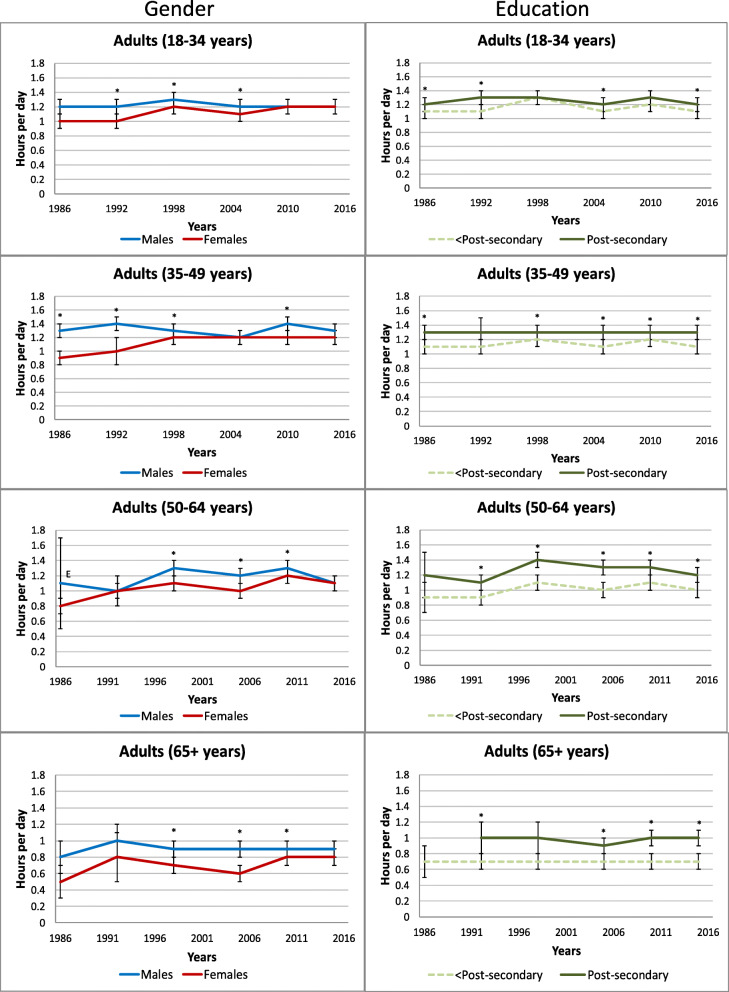
Self-reported average daily passive transport by gender and respondent education level within each age group**.** Error bars represent 95% confidence intervals around the mean. Stars (*) indicate differences at *p* < 0.05. GSS – Time Use Survey (1986, 1992, 1998, 2005, 2010, 2015)

Figure 7 displays self-reported passive travel time by gender and household education level within each adult age group in the GSS. Time spent in passive travel was often higher in men than women, but this gap appears to have narrowed with no significant differences in the most recent cycle (2015). Fairly consistent differences by education level were observed; those who were post-secondary graduates reported greater passive travel time than those who were not.

## Discussion

This is the first comprehensive examination of gender and education level differences in the sedentary behaviour of Canadians. Few significant differences were observed for accelerometer-measured total sedentary time. Consistent differences were; however, observed for time spent in specific sedentary activities, though they were often age- and survey- specific.

Previous research has found that among children and youth there is a consistent association between gender and objectively-measured sedentary time with boys being less sedentary than girls [30, 47]. There are also international data to suggest that the difference between boys and girls increases with age [47]. Among adults, however, no clear associations between gender and total objectively-measured sedentary time appear to exist [26]. Our findings generally agree with this evidence. Among children and youth, the most recent (2016–2017) cycle of the CHMS identified that boys were significantly less sedentary than girls. However, this difference was not observed in previous cycles except for youth in the 2007–2009 CHMS.

Among youth, systematic review evidence has identified an inconsistent association between gender and screen time [30, 48]. We found that among youth, historically when a summation of time spent in screen-specific behaviours (i.e., television + computer + video games) was used, boys were found to report more screen time than girls. This is similar to large international analysis of the HBSC study which found that boys report more screen time than girls [49]. While more recent data suggests these gender differences in screen time may be decreasing, it is important to consider that recent estimates were based either on a single global screen time question (CCHS) or additional types of sedentary behaviour included within questions or examples (e.g., HBSC computer chatting and electronic device time, CHMS smart phones). These changes make it difficult to ascertain if the gender gap is in fact closing or if reductions are the result of modifications to the measures.

Similar to our previous examination of these surveys [36], we found that different data sources tell different stories likely attributed to the types of questions asked, examples of sedentary activities used and the time reference for recall. For example, among youth, data from the HBSC suggests that boys watch more television than girls (in reference to a ‘usual weekday and weekend day’), however, this gender difference was not observed in the CCHS (2000–2014: typical week in past 3-months, 2015+: last 7 days). Furthermore, several cycles of the CHMS found the opposite was true (2007–2015 leisure time television including DVDs and videos vs. 2016+ asked respondents about free time television include DVDs, movies, internet videos over past 7 days). This is similar to what has been observed in population-based studies in the United States. American data from the 2001–2009 HBSC found that boys often reported significantly more television watching than girls [50]. Whereas, data from the 2007–2012 National Health and Nutrition Examination Survey (similar to the CCHS and CHMS) found no significant difference between boys and girls for television watching [51]. We also found that among youth, girls reported significantly greater use of electronic devices and online chatting than boys, whereas older data suggests that in the past boys engaged in more leisure computer use (possibly for other purposes).

Among adults 18–65 years, systematic review evidence suggests that female gender, in general, is negatively associated with total sitting, television and screen entertainment and passive travel when compared to male gender [26]. For older adults, there is a lack of consistent association between gender and sedentary behaviour (specifically total sedentary time and screen time) [33]. Our findings generally agree with this evidence. We found that total screen time was often significantly higher for younger men (18–34 and 35–49 years), but this was not observed for those aged 50–64 years or ≥ 65 years. Time-use data from the GSS found that men reported significantly more television viewing than women, but this was not consistently observed in the CCHS or CHMS. In fact, the CCHS found that older women watched more television than older men. The CCHS also identified that men reported more leisure computer use than women in all age groups except those aged 50–64 years where there were virtually no gender differences. These findings suggest a possible interaction between gender and age.

Our findings align with previous research on gender differences in leisure video game play [50, 52, 53], leisure reading [53–55], and passive/motorized travel [56–60]. We found that young men (12–17 and 18–34 years) spend significantly more leisure time playing video games than young women. There is evidence to suggest that gaming is less socially normative for girls/women, with women less likely to identify as ‘gamers’ and more likely to underreport their video game time and with boys more likely to report using video games for socialization [61–63]. Girls/women (≥ 12 years) tend to report more leisure time reading than boys/men. This may be a result of preferences for leisure activities, but may also be related to girls having greater intrinsic motivation for reading in childhood [64]. Among adults, time spent in passive travel was often higher in men compared to women, although more recently this gap appears to be closing. Evidence suggests that transit mode and use is highly shaped by gender [65]; women drive less, but are more likely to take public transit or be passengers in vehicles [66].

Based on comprehensive systematic review evidence, there appears to be a difference in the association between education/socio-economic status and sedentary behaviour across ages; with differences often dependent on whether sedentary behaviour is self-reported, device-assessed, or type-specific (e.g., screen time, reading) [25, 30]. Similar to what has been observed in the literature [25], we did not observe any consistent differences in objectively-measured sedentary time by household education level. In general, children, youth and adults from lower education households watched more television than those from higher education households; patterns were less clear in youth. Among children and youth, no statistically significant differences in leisure computer use were observed by household education level. Systematic review evidence has largely relied on television as a measure of screen-based behaviour and has found that parental education is inversely related to screen behaviour in cross-sectional studies especially in high-income countries [28, 29], but inconsistently associated in prospective studies [30]. Among adults, leisure computer use was greater among those with a higher household education. This is similar to what was observed in the UK Biobank Study, where college graduates averaged significantly more computer use than non-college graduates (1.35 h/day vs. 0.94 h/day) [67]. Similar to other international evaluations [68, 69], the GSS and CCHS found that adults with higher education or from households with a higher education reported higher levels of leisure time reading; though the relationship was more consistent in older age groups. Similar to other studies [59, 70], we found that post-secondary graduates often reported greater passive travel time than those who were not.

Previously, Liwander et al. stressed the need for gender and sex to be considered in the development of sedentary behaviour guidelines given that girls/women and boys/men often engage in different sedentary activities, that there are “gendered social and economic barriers that may influence sedentary behaviour” and that sex differences in the health outcomes associated with sedentary behaviour exist [71]. Results of our study bring increased awareness to the gender differences in the sedentary activities undertaken by Canadians. There is a continued need for both sex-stratified and gender-informed analyses when examining sedentary behaviour and for considering these differences in the design of interventions.

This study is not without limitations. First, the binary gender variable used to assess differences was largely based on biology. While it is useful for beginning to examine the difference between men and women, it is unidimensional (whereas gender is a multidimensional construct) and does not fully differentiate whether it is sex, gender or both that contribute to the differences [72]. While the data sources largely relied on measures of individual’s sex, we examined differences in sedentary behaviour recognizing that “… men and women are driven by the interplay of biologically and socially determined constructs of sex and gender” [73]. Both sex and gender will be collected in the CCHS and CHMS beginning in 2021. This paper provides a first step in identifying that sedentary behaviour may be influenced by gender. Future studies would benefit from a more in-depth examination of the social and cultural influences of gender on sedentary behaviour preferences. Secondly, we conducted a binary examination of sedentary behaviour by education level. While this binary education variable limits our ability to examine whether there is a gradient effect, it yielded greater power in analyses as often very few respondents per age group reported less than a high school education. Using a three-tiered variable (i.e., less than high school, high-school to less than post-secondary graduate, post-secondary graduate or greater) rendered many estimates un-releasable due to small sample sizes. Thirdly, while we have presented age-stratified results, it is possible that the composition of those in each age group across survey years differs due to a cohort effect. Fourthly, due to the differences in questions and response options between surveys, it was not possible to conduct formal time-series analyses across the type-specific sedentary activities. Finally, while all of the surveys employed are designed to be nationally representative, they present with variable response rates (e.g., CCHS: ~ 67–88% [74], CHMS: ~ 35–42% [activity monitor] [75, 76], GSS: ~ 38–79% [40]) with rates generally declining over time.

## Conclusions

While few differences were found for total objectively-measured sedentary time, gender and education differences in type-specific sedentary behaviour were more prevalent. Cross-survey data consistently identified that male gendered individuals engaged in more video game play, leisure screen time, and passive travel, while female gendered individuals engaged in more leisure reading. Those with a higher education or household education reported more leisure reading and passive travel. Education differences in screen time were often age dependent, with leisure computer use greater in higher education groups in adults only and leisure television watching generally higher with lower education in children and adults, but not youth. This information is valuable as it provides further information on possible target audiences and behaviours for sedentary behaviour policies and interventions. Future work is needed to further understand factors contributing to these differences.

## Data Availability

The datasets analysed during the current study are available to researchers pending proposal approval through the Research Data Centres (RDC) Program at Statistics Canada (https://www.statcan.gc.ca/eng/rdc/index).

## Abbreviations

CCHS: Canadian Community Health Survey
CHMS: Canadian Health Measures Survey
CI: Confidence interval
GSS: General Social Survey
HBSC: Health Behavior in School-Aged Children
